# Identifying Research Priorities in Early Psychosis: A Collaborative Approach to Shaping the Future of Early Psychosis Clinical Trials in Australia

**DOI:** 10.1111/eip.70170

**Published:** 2026-04-07

**Authors:** Amity E. Watson, Stephen J. Wood, Ellie Brown, Isabel Zbukvic, Joanna Fitzsimons, Deanna De Cicco, Karine Fernandez, Georgia Williams, Andrew Thompson

**Affiliations:** ^1^ Orygen, the National Centre of Excellence in Youth Mental Health Parkville Victoria Australia; ^2^ Centre for Youth Mental Health The University of Melbourne Parkville Victoria Australia; ^3^ School of Psychology University of Birmingham Edgbaston UK; ^4^ Centre for Mental Health and Brain Sciences, School of Health Sciences Swinburne University of Technology Melbourne Australia

**Keywords:** early intervention, health services research, lived experience, psychotic disorders, research prioritisation, stakeholder participation

## Abstract

**Introduction:**

Psychotic disorders are major contributors to disability and mortality, disproportionately affecting young people. Despite advances in early psychosis care, there are still barriers to treatment, suboptimal outcomes, and limited therapeutic options. Given limited funding for psychosis research, priority setting that incorporates perspectives from across the sector, but especially those with lived experience, is vital for directing resources. This paper outlines the Australian Early Psychosis Collaborative Consortium's process for identifying priorities to guide early psychosis clinical trial research in Australia.

**Methods:**

Qualitative data were collected via online surveys and stakeholder workshops with people with lived experience, carers, clinicians and researchers. Content analysis was conducted, followed by iterative refinement and ranking of priority research questions across multiple stages.

**Results:**

Six broad themes and 55 research questions were identified through both the initial survey and stakeholder consultations, culminating in ten research questions for ranking in the final poll. The top‐ranked priority was identifying treatments with the most enduring benefits for individuals with early psychosis. Other priorities included: identifying effective treatments for negative symptoms; addressing trauma in psychosis risk and treatment; focusing on supports for neurodevelopmental conditions; understanding and targeting risk factors to prevent psychosis onset; translation of research findings to clinical care; considerations of cultural diversity; and effective support for people leaving early psychosis services.

**Conclusion:**

These findings reaffirm the core objective of specialised early intervention for psychosis: to support long‐term recovery and improved outcomes. Identified priorities offer direction for future research and resource allocation that reflects diverse stakeholder perspectives.

## 1 Introduction

Psychotic disorders can have a profound impact on affected individuals and their families (Estrade et al. 2023; Fusar‐Poli et al. 2022) leading to significant impairments in functioning, increased rates of unemployment, social isolation, and elevated mortality risk (Correll et al. 2022; Dong et al. 2019; Fulford and Holt 2023; Hakulinen et al. 2019). In Australia, the annual societal cost of psychosis, including healthcare expenses and lost productivity, exceeds $6 billion AUD (Sweeney et al. 2016), highlighting psychosis as a major public health concern and the urgent need for advances in research to better inform prevention and early intervention.

Australia has played a leading role in pioneering specialised early intervention programs for individuals experiencing first‐episode psychosis (FEP) and those at risk of developing it (Malla and McGorry 2019). The primary goal of early intervention is to alter the course of psychotic illness and improve long‐term outcomes by: (1) preventing the transition to psychosis in those considered at high‐risk, and (2) minimising the duration of untreated psychosis—a critical factor in determining outcomes (Howes et al. 2021; Salazar de Pablo et al. 2024)—through the delivery of high‐quality evidence‐based treatments (Early Psychosis Guidelines Writing Group and EPPIC National Support Program 2016). These models of care have demonstrated effectiveness for better clinical and functional outcomes, including symptom reduction and decreased hospitalisation rates, and improved global, social and occupational functioning and quality of life (Correll et al. 2018; Puntis et al. 2020). However, many individuals with early psychosis still face significant delays in accessing treatment (Murden et al. 2024), and real‐world outcomes remain deeply inadequate, failing to provide sustained recovery and functional improvement over the long‐term (Hansen et al. 2023). This is in part due to inconsistent and fragmented implementation of early intervention models (O'Connell et al. 2021), and heterogeneity of treatment response (Griffiths et al. 2022). Knowledge gaps persist in both the development of novel therapeutic interventions and personalised treatment approaches, highlighting a critical need for high‐quality, targeted research and clinical trials that can be effectively translated into practice.

Given the resource constraints in mental health research (Batterham et al. 2016; Yung et al. 2023) priority setting is an essential strategy to ensure funding is directed toward the most pressing issues in early psychosis care. Prioritising research questions can help focus efforts on interventions with the greatest potential for impact, especially when shaped by diverse stakeholder perspectives. To align with real‐world needs, research must incorporate insights from key stakeholders (Gulliver et al. 2022), individuals with lived experience (LE; i.e., those with a personal experience of psychosis, carers and family members) and early psychosis clinicians. Stakeholder‐driven research not only enhances the relevance and applicability of findings but also improves engagement, recruitment, and study completion rates (Ennis and Wykes 2013). Furthermore, LE participation in both research and care can provide deeper insight into the experience of psychosis and help reduce stigma (Sibeoni 2023). Although research priority‐setting exercises have been undertaken in broader mental health contexts (Güell et al. 2023; Mei et al. 2020), there is a notable gap in psychosis‐specific initiatives, with little work published in this area (Peltzer‐Jones et al. 2019; Sher et al. 2024), and only one to our knowledge including LE perspectives (Renwick et al. 2022).

To address these challenges, and given the urgent need to advance psychosis research, strategic collaborative efforts are essential. The Australian Early Psychosis Collaborative Consortium (AEPCC; Thompson et al. 2023) has established a national early psychosis lived experience network (LEN), an early psychosis clinical quality registry (CQR), and a clinical trials and translation network (CTTN). This collaborative consortium focuses on improving outcomes for early psychosis. By bringing together LE individuals, clinicians and researchers, AEPCC aims to support the development of clinical trials that are both scientifically rigorous and aligned with the specific needs and priorities of the early psychosis community. The CTTN and CQR in combination have strengthened the connection between research teams and clinical sites across Australia, the benefits of which will be a significantly reduced time required to complete a study from inception to results, ensuring faster translation of findings into practice and enabling more responsive and adaptive approaches to treatment in the field (Nemeh et al. 2022; Thompson et al. 2023).

This paper outlines the research priority‐setting process conducted by AEPCC to identify key areas for clinical trials in early psychosis. Through a structured, multi‐stage approach involving surveys and stakeholder consultations, this initiative aims to direct research efforts toward the most urgent needs in early psychosis care, ultimately improving outcomes for people affected by psychosis.

## 2 Method

### 2.1 Participants

Participants included individuals from three stakeholder groups across the early psychosis sector in Australia: (1) people with lived experience of psychosis, carers and family members (referred to collectively as LE here); (2) clinicians and professionals supporting people with early psychosis; and (3) early psychosis researchers. Surveys were online; most participants were from Australia. The LE group included individuals at various stages of illness.

### 2.2 Design

**FIGURE 1:**
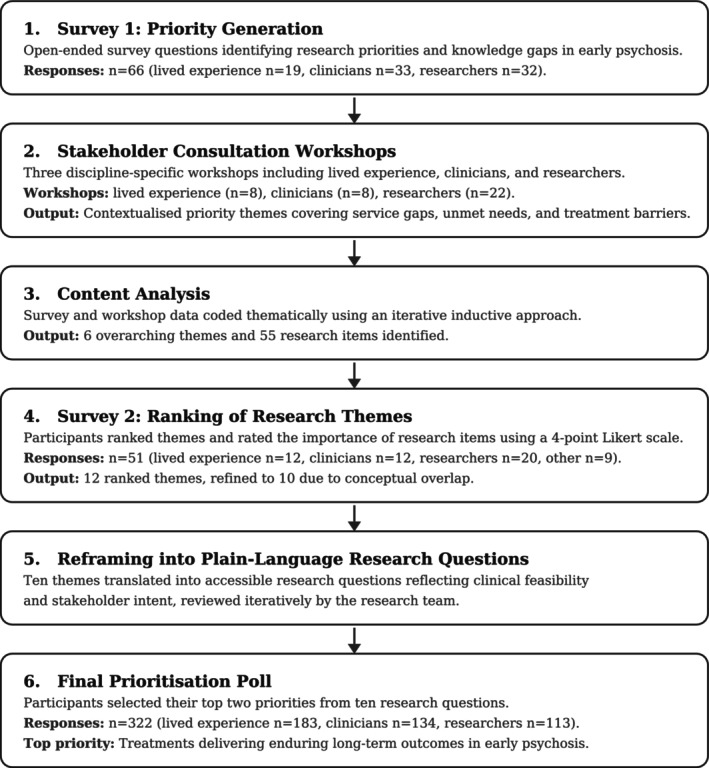
Process and outcomes of multi‐stage, stakeholder‐informed research prioritisation. At Survey 1 and the final prioritisation poll, respondents who selected 'other' were provided a free‐text box; responses were reviewed and recoded into one of the three predefined stakeholder categories where appropriate. ‘Other’ responses included a mix of carers, family members, friends, and advocates for someone with lived experience of psychosis, and peer workers; these were recoded as lived experience. At the ranking survey (Step 4), recoding was not possible as no free‐text option was provided; the 'other' category is therefore retained at this step. Participants could identify across multiple stakeholder groups; group totals therefore exceed the number of respondents. Key outputs included the identification of six overarching research themes (see Table 1), 55 research items (see Table 2), and 10 final research questions (see Table 4). The top‐rated priority focused on identifying treatments that provide the most enduring long‐term outcomes for individuals with early psychosis.

Data collection occurred from March 2022 to December 2023 through a staged, mixed‐methods research‐priority setting process informed by the ACTA Research Prioritisation Framework (Nasser et al. 2013) and in accordance with established guidance for good practice in health research priority setting, which emphasises transparency, inclusiveness, use of mixed qualitative and quantitative methods, and iterative refinement rather than adherence to a single prescriptive framework (Deering et al. 2023). Six key stages were undertaken: an initial open‐ended stakeholder survey; stakeholder consultation workshops; content analysis; a ranking survey; reframing of priorities into research questions; and a final prioritisation poll (Figure 1).

### 2.3 Recruitment

Links to surveys were distributed through the AEPCC newsletter, directly to AEPCC members and promoted across social networks, early psychosis services (e.g., *headspace*), and national and international conferences. Participants for stakeholder consultation were recruited via direct invitation and an expression of interest form in Survey 1.

### 2.4 AEPCC Working Group and Advisory Committees

The priority setting process was led by the AEPCC working group, comprising representatives with LE, clinical, research, and leadership expertise. AEPCC's advisory council and CTTN Scientific Committee provided ongoing feedback on methodology and scope.

### 2.5 Stakeholder Workshops

Separate workshops for each stakeholder group explored perspectives on knowledge gaps, research strengths, weaknesses, and key priorities. Participants were guided by questions including: What are the major gaps in our understanding of early psychosis treatment? What are the strengths and weaknesses of early psychosis research in Australia? What should the research priorities be? Clinician discussions also focused on identifying urgent, practical research questions with direct relevance to patient care, while the researcher consultation built on previous priority‐setting efforts (McGorry 2015) to evaluate relevance of past priorities and identify emerging gaps.

### 2.6 Content Analysis

Qualitative data from Survey 1 and stakeholder consultation workshops were collated and analysed using content analysis (Sandelowski 2000). This inductive approach enabled research priorities to emerge from participant responses rather than being constrained by researcher assumptions.

Two members of the research team (E.B., G.W.) independently coded the open‐ended survey responses and supplemented these with relevant data from workshop transcripts. Similar responses were collapsed where appropriate, with coding conducted iteratively to group items into higher‐order categories.

To validate the results, preliminary themes were reviewed by AEPCC's working group and CTTN Scientific Committee, confirming that categories reflected the breadth of stakeholder input and captured both expected and emerging priorities.

### 2.7 Ranking Survey

A second survey was conducted to rank the themes and items drawn from the content analysis. Participants were asked to rank six themes (Table 1) in order of importance, with 1 being the most important and 6 being the least important. Participants were then asked to rate the importance of each of 55 individual items on a 4‐point scale (1 = not important, to 4 = very important).

**TABLE 1:** Topics identified by content analysis for inclusion in Survey 2; participants ranked in order of importance from 1 to 6.

Theme 1: Causes and risks associated with developing psychosis
Theme 2: Diagnosis and treatment of early psychosis
Theme 3: Models of care for early psychosis
Theme 4: Comorbidity and complexity in early psychosis (e.g., physical health and early psychosis, trauma and early psychosis)
Theme 5: Impact and long‐term outcomes of early psychosis
Theme 6: Other areas of research in early psychosis

### 2.8 Final Prioritisation Poll

In consultation with AEPCC's LE and CTTN Scientific Committees, the top themes to emerge from the ranking survey were reframed into research questions using simplified and more accessible language, which then formed the items to be included in the final poll.

## 3 Results

The outcomes of each stage in the research prioritisation process, including response rates and participant composition at each step, and how research questions were refined and ranked are summarised in Figure 1.

### 3.1 Lived Experience Consultation Workshop

Some LE participants expressed a desire for greater understanding of the neurobiological mechanisms underlying psychosis, highlighting the need for research into its causes and risk factors. Service model challenges were a major theme, particularly gaps in the transition from youth to adult care and the lack of coordination between these systems. The physical design of clinics was also seen as a barrier, with calls for more inclusive and accessible environments.

Participants emphasised shared decision‐making in treatment and suggested education initiatives to improve awareness among service users, carers, and clinicians.

Concerns were also raised about the long‐term consequences of psychosis, difficulties in regaining confidence and achieving functional recovery. This underscores the need for research into strategies that enhance social and functional outcomes while reducing the lasting impact of psychosis.

### 3.2 Clinical Consultation Workshop

A major concern for clinicians was the lack of continuity in care, with funding constraints often forcing young people to seek treatment through unaffordable private services post‐discharge. Clinicians noted challenges in culturally safe and recovery‐oriented discharge planning, especially for First Nations populations, emphasising the need for tailored models of care.

Lack of psychoeducation was flagged as a critical gap, particularly for young people without supportive social networks. Furthermore, social isolation was identified as a significant issue. Managing comorbid conditions, such as the relationship between sexual trauma and psychosis, was another theme, with participants advocating for integrated, trauma‐informed services.

Clinicians raised concerns about medication management, a lack of research on young people's experiences with medication, long‐term effects, and withdrawal processes.

Finally, the potential of technology to improve engagement and care delivery was suggested, with clinicians noting that in their experience young people preferred digital tools and text‐based communication.

### 3.3 Consultation With Researchers

Researcher discussions centred on the need to understand variability in treatment response and the role of social determinants, such as homelessness, in shaping outcomes. Participants stressed the importance of including marginalised populations, particularly First Nations communities, and called for culturally diverse research frameworks.

Matching treatment to individual needs remained a key theme, with advocates highlighting innovative approaches such as transdiagnostic cognitive behavioural therapy and trauma‐informed care. Particular focus was placed on treating comorbid trauma, depression, and negative symptoms, and integrating physical and sexual health into treatment plans.

Researchers were concerned about fragmented care pathways and emphasised sustained monitoring post‐discharge. Suggested interventions included self‐management strategies, relapse prevention initiatives, digital tools, and education about early warning signs.

Researchers also discussed the complexity of psychosis presentations, especially in underserved regions, calling for comprehensive treatment packages tailored to the unique needs of remote and diverse populations, proper screening tools and further investigation into culturally sensitive treatment responses. Technology‐driven interventions were also suggested to improve engagement and accessibility.

### 3.4 Emerging Themes and Ranked Research Priorities

Table 1 summarises the six overarching research themes identified through content analysis and included in the ranking survey, while the importance ratings for 55 research items also included in the ranking survey are presented in Table S1. Nine research priorities were rated as “very important” by more than 60% of respondents and are summarised in Table 2.

**TABLE 2:** Summary of highest‐ranked research priorities from the stakeholder ranking survey. Priorities are ordered by the proportion of respondents rating each item as “Very important”.

Rank	Research priority	Very important (%)	*n*
1	Translation of research findings into clinical practice	74.5	51
2	Understanding and treating trauma in early psychosis	72.5	51
3	Access to evidence‐based treatment for early psychosis	68.8	48
4	Personalised treatment for early psychosis	66.0	53
5	Culturally appropriate models of care for early psychosis	65.4	52
6	Social determinants of health and psychosis risk	64.8	54
7	Improving social connection and life skills	62.7	51
8	Treatments for negative symptoms in early psychosis	62.3	53
9	Risk factors associated with developing early psychosis	61.1	54

*Note: n* represents the number of respondents who rated each item. All 55 research items included in the ranking survey are reported in Table S1. For clarity, this table presents the nine highest‐ranked items, defined as those rated as “very important” by more than 60% of respondents.

Table 3 presents the broader thematic areas that emerged across the priority‐setting process, including survey responses and stakeholder workshops, prior to the final poll. The final ten research themes and their corresponding stakeholder‐informed research questions included in the prioritisation poll, ordered by overall ranking, can be seen in Table 4.

**TABLE 3:** Top 12 themes to emerge from responses to Survey 2.

Ranking	Theme
1	Translation of research findings into clinical care
2	Understanding and treating trauma and early psychosis
3	Culturally appropriate models of care for early psychosis, e.g., social and emotional wellbeing models of care
4	Treatments for negative symptoms (e.g., reduced motivation, reduced drive, and enjoyment)
5	Non‐medication treatment for early psychosis
6	Improving social connection and life skills including culturally specific groups
7	Personalised treatment for early psychosis
8	What are the risk factors associated with relapse in early psychosis
9	Long‐term effects of antipsychotic medication
10	Working with First Nations young people and their families
11	Understanding and treating neurodevelopmental impacts and early psychosis
12	Access to evidence‐based treatment for early psychosis, fidelity of care

**TABLE 4:** Final ten themes and their corresponding research questions included in the prioritisation poll, according to final ranking.

Overall ranking	Theme	Corresponding research question
1	Predicting sustainable outcomes (e.g., symptoms, quality of life, general mental health, and functioning)	What early psychosis treatments deliver the best long‐term benefits?
2	Treatments for negative symptoms (e.g., reduced motivation, reduced drive and enjoyment)	What are the best ways to support people with early psychosis experiencing “negative symptoms”, like disinterest in social connections or disconnection from feelings?
3	Understanding and treating trauma and early psychosis	How can we best support young people with early psychosis who have experienced trauma?
4	Treatment refractory psychosis	Can we develop successful new treatments for people experiencing early psychosis for whom usual care hasn't helped?
5	Understanding and treating neurodevelopmental impacts and early psychosis	What are the best treatments for people with EP who have a history of childhood neuro development conditions such as autism or ADHD?
6	Understanding the risk factors associated with developing early psychosis (e.g., genetic, environmental, epigenetic, pathogenic mechanisms, gender, biological, microbiome, substance use, trauma)	How can we treat risk factors to prevent the onset of psychosis?
7	Long‐term effects of antipsychotic medication	What are the positive and negative impacts of long‐term treatment with antipsychotic medications?
8	Translation of research findings into clinical care	How best to apply findings from research studies to clinical treatments?
9	Culturally appropriate models of care for early psychosis, e.g., social and emotional wellbeing models of care	What are the best clinical ways to support young people with EP who are from diverse cultural backgrounds
10	What happens next? Post‐early psychosis service/treatment care planning	What are the best ways to support people leaving early psychosis services?

**FIGURE 2:**
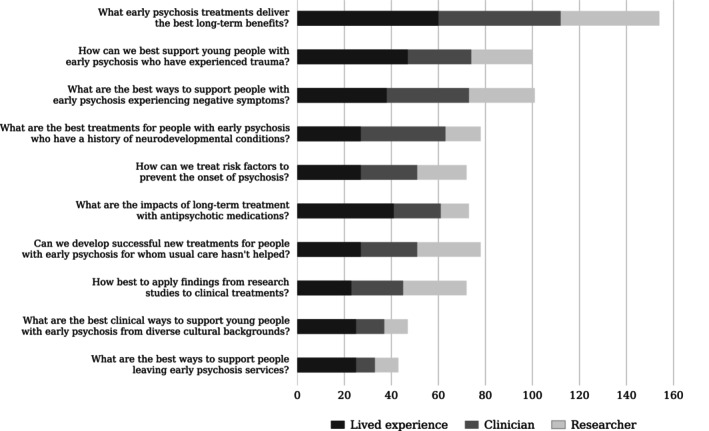
Results from prioritisation poll in which participants voted for their top two priorities for early psychosis research. Multiple responses were counted for participants who identified as belonging to more than one stakeholder group.

Figure 2 shows the distribution of votes from the final prioritisation poll, indicating the relative importance of early psychosis research priorities across stakeholder groups.

## 4 Discussion

We have detailed here AEPCC's process to identify key priorities for clinical trials research in Australia's early psychosis sector, grounded in the real‐world needs of individuals with lived experience of psychosis, families and carers, clinicians, and researchers. While stakeholder‐driven priority setting is gaining traction in mental health research broadly (Güell et al. 2023), few initiatives have focused specifically on early psychosis (Renwick et al. 2022). To our knowledge, this is the first effort to define research priorities for early psychosis in Australia, providing a crucial foundation for future clinical trials that align with the needs of those most affected.

The process culminated in a list of 10 research questions intended to guide future efforts. Notably, the top ranked priority by a clear margin, and across all groups, was determining which treatments offer the most enduring benefits for individuals with early psychosis. This aligns with the core goal of early intervention: to improve outcomes and alter the trajectory of psychosis through intensive treatment, prevent relapse, and promote sustained recovery from FEP (McGorry et al. 2007). However, it also highlights one of the known weaknesses in the existing model: that improvements are not always maintained over time (Hansen et al. 2023). A major challenge for early intervention is that the care offered is time‐limited, making longer‐term outcomes and post‐discharge pathways difficult to track. The point of referral after early intervention can significantly shape a young person's long‐term recovery and overall well‐being. While adult mental health care may offer ongoing, specialised support suitable for the complexities of psychosis, there are many factors that may lead to disengagement following the transition from adolescent to adult services (Arango et al. 2022). Conversely, many young people are discharged from specialist youth services to primary care or private practice clinicians (O'Donoghue et al. 2023), where lower‐intensity treatment may create gaps in care. This observation also aligns with the 10th‐ranked priority in the final priorities poll: identifying how best to support individuals leaving early psychosis services. While it received fewer votes overall than the top priority, it nonetheless emerged as a consistent concern across all stakeholder groups during the workshop discussions. A promising direction for clinical trials could be identifying subgroups at higher risk of poorer outcomes, who may benefit from extended specialised early intervention, more tailored treatment strategies (O'Donoghue et al. 2023; Puntis et al. 2018), or from supported self‐management interventions after leaving specialised care (Milton et al. 2024).

The second and third highest ranked priorities related to understanding and treating both negative symptoms and trauma. This finding aligns with previous priority‐setting work (Renwick et al. 2022), which emphasised adverse childhood experiences and the significance of trauma‐informed care as a critical area for development in early psychosis services. It is recognised that trauma can contribute to the onset and persistence of psychotic symptoms and, despite evidence that trauma‐informed approaches can improve engagement and outcomes such as reduced symptom severity and relapse rates (Bendall et al. 2021), targeted, evidence‐based interventions for young people with psychosis and a history of trauma are lacking (Hardy et al. 2023). Similarly, the need to address negative symptoms was highlighted in both Renwick et al. (2022) and our findings. Negative symptoms—including diminished motivation, social withdrawal, and emotional blunting—are among the most disabling aspects of psychotic disorders and are strongly linked to poor functional recovery (Correll and Schooler 2020). Despite such impact, this symptom domain remains difficult to treat; there are currently no recommended first‐line pharmacological treatments, and psychosocial interventions only show modest benefits (Cella et al. 2023). Improving treatments for negative symptoms could support functional engagement, social reintegration, quality of life and long‐term outcomes, thus reinforcing the need for targeted interventions in this domain.

The remaining priorities, each closely ranked with similar response rates, encompassed a broad range of concerns. Among them was identifying the most effective treatments for individuals with early psychosis who have a history of childhood neurodevelopmental conditions (e.g., autism or ADHD), as well as pinpointing and addressing risk factors that may precipitate psychosis. The importance of mitigating psychosis risk was similarly highlighted in the study by Renwick et al. (2022). Despite earlier evidence of a benefit for community and school‐based detection programs to reduce the transition to full‐threshold psychosis (Fusar‐Poli et al. 2020), a recent meta‐analysis (Minichino et al. 2025) concluded that there are in fact no interventions proven to actually lower risk. This gap highlights the urgent need for innovative strategies that can prevent the onset of early psychosis by targeting underlying risk factors, while also recognising the importance of accounting for the heterogeneity within high‐risk populations.

Findings suggest that research should also focus on long‐term negative and positive effects of antipsychotic use, development of new treatments for individuals who do not respond to standard care and translating emerging research findings into effective clinical interventions. While advancements in psychosis treatment continue, the translation of research into routine clinical practice remains slow, often impeded by limited funding, inadequate training, and structural barriers within healthcare systems (e.g., Darker et al. 2023). Bridging this gap requires not only investment in new clinical trials but a stronger emphasis on implementation science frameworks to help identify barriers, enhance intervention fidelity, and support the sustained adoption of effective treatments (Zbukvic et al. 2022). Furthermore, embedding lived experience perspectives and clinician expertise throughout this process is essential to ensuring new interventions are relevant, practical, and widely accessible (Hawke et al. 2024). A co‐designed, collaborative approach involving individuals with psychosis, their families, and frontline clinicians will better align research with real‐world needs, driving the development of evidence‐based, person‐centred treatment innovations.

Finally, identifying the most effective clinical approaches for young people from diverse cultural backgrounds also emerged as a priority. This reflects the complex interplay between cultural identity, help‐seeking behaviours, and systemic barriers that can limit access to appropriate care. Factors such as language, stigma, culturally incongruent service models, and historical mistrust of healthcare systems may all contribute to disparities in engagement and outcomes (Luu et al. 2023; Ocloo et al. 2021). Ensuring that clinical approaches are culturally responsive and tailored to the specific needs of diverse populations is essential for improving access, equity, and the overall effectiveness of early psychosis interventions (Rathod et al. 2023).

### 4.1 Challenges and Areas for Improvement

Recent reviews of health research priority‐setting exercises highlight substantial heterogeneity in methods, frequent under‐reporting of analytic procedures, and limited evaluation of downstream impact (Deering et al. 2023). In this context, key strengths of the present exercise include transparent reporting of each stage, explicit inclusion of lived experience alongside clinical and research stakeholders, and iterative refinement of priorities into answerable research questions. Although a formal process such as the James Lind Alliance (JLA; James Lind Alliance 2021) was not adopted, our approach drew on core principles common to established priority‐setting frameworks such as JLA, including transparency, inclusivity, and iterative stakeholder involvement, while also being informed by broader guidance for good practice in health research and clinical trials priority setting (Australian Clinical Trials Alliance 2020). Adoption of a full JLA partnership model represents a potential future direction as the capacity and national engagement of AEPCC expands.

A key challenge was the smaller number of participants in Survey 1, which may have constrained the diversity of perspectives captured at this stage, particularly from underrepresented groups such as First Nations peoples and those from rural or regional areas. Recruitment was primarily through existing early psychosis networks, limiting reach beyond mainstream services. Strategies to broaden participation will be used in future rounds, including partnership with Aboriginal and Torres Strait Islander community‐led health organisations and targeted outreach through regional youth mental health networks. Refining survey language and format may also improve completion rates. Nevertheless, representation across stakeholder groups was reasonably well balanced, and priorities identified in Survey 1 were explored in greater depth during the workshops, iteratively refined across subsequent stages, and further strengthened through validation of emerging themes. The relevance and representativeness of the findings were additionally supported by strong stakeholder engagement in the final prioritisation poll. These limitations reflect challenges commonly identified in the priority‐setting literature and point to important directions for methodological development in future work.

### 4.2 Next Steps and Future Research

Beyond informing academic research agendas, the priorities identified here are intended to directly inform future clinical trials and translation initiatives, particularly those supported by AEPCC. The refinement of stakeholder identified priorities into answerable research questions provides a platform for structured engagement with funders, policymakers, and service leaders, including cross‐referencing these priorities with existing funding portfolios to support coordinated investment in areas of highest stakeholder need. As AEPCC's CTTN and CQR continue to mature, these priorities can be operationalised through pragmatic or adaptive platform trials embedded within real‐world services, as well as through registry‐based and data‐linkage approaches. Together, these approaches have the potential to support a learning health system model in which evidence generation and service improvement occur in parallel, enabling identification of treatments that deliver sustained functional and quality‐of‐life benefits (Heinssen and Azrin 2022).

## 5 Conclusion

These findings reinforce the core aim of specialised early intervention for psychosis services to improve long‐term outcomes and highlight a shared consensus on the importance of addressing trauma and negative symptoms in early psychosis. Together, these priorities strengthen the case for targeted investment in research and clinical practice, helping ensure that future research efforts and resource allocation align with the needs identified by diverse stakeholder groups.

## Funding

This work is part of the AEPCC initiative, which is supported by the Wellcome Trust (218254/Z/19/Z).

## Conflicts of Interest

The authors declare no conflicts of interest.

## Supporting information


**Table S1:** Importance ratings for all research items included in the ranking survey.

## Data Availability

The data that support the findings may be available on request from the senior author. The data are not publicly available due to privacy restrictions.
