# Supplementary material for: Identifying Research Priorities in Early Psychosis: A Collaborative Approach to Shaping the Future of Early Psychosis Clinical Trials in Australia
Source: Early Interv Psychiatry. 2026 Apr 7;20(4):e70170. doi: 10.1111/eip.70170 (PMC13054207; doi:10.1111/eip.70170)
Supplement: Supplementary file 1 — Table S1: Importance ratings for all research items included in the ranking survey. [file EIP-20-0-s001.docx]

# Supplementary Table S1. Importance ratings for all research items included in the ranking survey

| Research priority | n | Very important (%) | Quite important (%) | Low importance (%) |
| --- | --- | --- | --- | --- |
| 01. Understanding what early psychosis is and what causes it. | 54 | 53.7 | 27.8 | 18.5 |
| 02. Understanding the risk factors associated with developing early psychosis (e.g. genetic, environmental, epigenetic, pathogenic mechanisms, gender, biological, microbiome, substance use, trauma) | 54 | 61.1 | 25.9 | 13.0 |
| 03. How do social determinants of health (e.g., social/community context, health care access, education access, economic stability) contribute to the risk of developing early psychosis (and how could this be avoided) | 54 | 64.8 | 22.2 | 13.0 |
| 04. How can we best predict and identify those at risk of developing early psychosis | 54 | 46.3 | 35.2 | 18.5 |
| 05. What are the risk factors associated with relapse in early psychosis | 54 | 59.3 | 35.2 | 5.6 |
| 06. Screening and diagnosis of ultra-high risk for psychosis (e.g. at clinical risk for developing psychosis) | 53 | 26.4 | 41.5 | 32.1 |
| 07. Treatment for ultra-high risk for psychosis (including early medical treatment) | 53 | 37.7 | 32.1 | 30.2 |
| 08. Screening and diagnosis of first episode psychosis | 52 | 46.2 | 42.3 | 11.5 |
| 09. Treatments for positive symptoms (e.g. hallucinations, delusions and confused thinking) | 52 | 46.2 | 46.2 | 7.70 |
| 10. Treatments for negative symptoms (e.g. reduced motivation, reduced drive and enjoyment) | 53 | 62.3 | 35.8 | 1.9 |
| 11. Non-medication treatments for early psychosis | 53 | 58.5 | 37.7 | 3.8 |
| 12. New medication treatments for early psychosis | 53 | 32.1 | 37.7 | 30.1 |
| 13. Better management of medications, including side effects | 53 | 50.9 | 35.8 | 13.2 |
| 14. Personalised treatment for early psychosis | 53 | 66.0 | 22.6 | 11.3 |
| 15. Targeted treatment for different stages of early psychosis | 53 | 49.1 | 30.2 | 20.8 |
| 16. Treatment targeting social determinants (e.g., social/community context, health care access, education access, economic stability) | 53 | 52.8 | 32.1 | 15.1 |
| 17. Technology-based treatments (e.g. virtual reality, neurofeedback) | 53 | 13.2 | 30.2 | 56.6 |
| 18. Novel non-medical approaches to treatments (e.g. sensory modulation, | 53 | 22.6 | 39.6 | 37.7 |
| 19. New pragmatic clinical trial methodologies e.g. platform and registry trials, biological samples | 53 | 17.0 | 45.3 | 37.7 |
| 20. Family (and carer) therapy and the impact of family dynamics | 53 | 43.4 | 45.3 | 11.3 |
| 21. New and creative psychosocial treatments (e.g. group programs) | 52 | 36.5 | 46.2 | 17.3 |
| 22. Access to evidence-based treatment for early psychosis, fidelity of care | 48 | 68.8 | 27.1 | 4.2 |
| 23. Co-designed services for early psychosis | 52 | 48.1 | 30.8 | 21.1 |
| 24. Integration of primary care in early psychosis treatment (e.g. GPâ€™s) | 52 | 40.4 | 40.4 | 19.2 |
| 25. Health utilisation and pathways through care | 52 | 40.4 | 32.7 | 26.9 |
| 26. Role of peer support and peer workers in both ultra-high risk and first episode psychoisis | 52 | 48.1 | 34.6 | 17.3 |
| 27. Role of families and carers in care for early psychosis | 52 | 46.2 | 42.3 | 11.5 |
| 28. Support for families and carers | 52 | 46.2 | 46.2 | 7.7 |
| 29. Models of care in regional/rural/remote areas | 52 | 59.6 | 25.0 | 15.4 |
| 30. Culturally appropriate models of care for early psychosis e.g. social and emotional wellbeing models of care | 52 | 65.4 | 28.8 | 5.8 |
| 31. What happens next? Post-early psychosis service/treatment care planning | 52 | 57.7 | 26.9 | 15.4 |
| 32. Qualitative/personal experience of care | 40 | 50.0 | 27.5 | 22.5 |
| 33. Psychosocial interventions (e.g. | 52 | 28.8 | 53.8 | 17.3 |
| 34. Engagement in early psychosis care | 52 | 44.2 | 40.4 | 15.4 |
| 35. Shared decision-making in early psychosis care | 52 | 55.8 | 34.6 | 9.6 |
| 36. Home based care, preventing hospitalisations, safer hospitalisations | 52 | 48.1 | 42.3 | 9.6 |
| 37. Involuntary treatment within early psychosis care and custodial settings | 52 | 25.0 | 42.3 | 32.7 |
| 38. Understanding and treating physical and sexual health comorbidities (including diet, smoking, physical activity, sleep) and early psychosis | 51 | 47.1 | 33.3 | 19.6 |
| 39. Understanding and treating trauma and early psychosis | 51 | 72.5 | 19.6 | 7.8 |
| 40. Understanding and treating alcohol and other drug use and early psychosis | 51 | 52.9 | 37.3 | 9.8 |
| 41. Understanding and treating neurodevelopmental | 51 | 43.1 | 41.2 | 15.7 |
| 42. Understanding and treatments for diagnostic complexity (e.g intellectual disability/ASD/ADHD, physical health, neurodiversity, epilepsy, other mental health presentations) | 51 | 49.0 | 37.3 | 13.7 |
| 43. Treatment refractory psychosis | 51 | 52.9 | 31.4 | 15.7 |
| 44. Identification of and interventions for neurocognitive impairment | 51 | 41.2 | 45.1 | 13.8 |
| 45. Understand variations in treatment responses and outcomes (e.g. gender, culturally and lingustically diverse communities, First Nations communities) | 51 | 52.9 | 33.3 | 13.7 |
| 46. Long-term effects of antipsychotic medication | 51 | 54.9 | 39.2 | 5.9 |
| 47. Predicting sustainable outcomes (e.g. symptoms, quality of life, general | 51 | 52.9 | 39.2 | 7.8 |
| 48. Improving social connection and life skills inc. culturally specific groups | 51 | 62.7 | 27.5 | 9.8 |
| 49. Qualitative/personal understanding of impact of psychosis on a young person (e.g. shame, stigma, identity) | 50 | 52.0 | 30.0 | 18.0 |
| 50. Translation of research findings into clinical care | 51 | 74.5 | 21.6 | 3.9 |
| 51. Translation of research findings into community settings (e.g. multicultural settings, education settings etc.) | 51 | 51.0 | 39.2 | 9.8 |
| 52. Culturally sensitive and appropriate education and information for families and carers | 51 | 49.0 | 37.3 | 13.8 |
| 53. Working with First Nations young people and their families | 51 | 56.9 | 35.3 | 7.8 |
| 54. Working with young people and | 51 | 54.9 | 33.3 | 11.8 |
| 55. Stigma reduction and education about psychosis â€“ across healthcare settings, family, education etc. | 51 | 58.8 | 25.5 | 15.7 |
